# Punctuated Equilibrium: COVID and the Duty to Teach for Adaptive Expertise

**DOI:** 10.5811/westjem.2021.11.55268

**Published:** 2022-01-03

**Authors:** Chris Merritt, Sally A. Santen, Stephen John Cico, Margaret Wolff, Martin Pusic

**Affiliations:** * Alpert Medical School of Brown University, Department of Emergency Medicine, Providence, Rhode Island; † Virginia Commonwealth University School of Medicine, Department of Emergency Medicine, Richmond, Virginia; ‡ University of Cincinnati, Departments of Emergency Medicine and Medical Education, Cincinnati, Ohio; § Indiana University School of Medicine, Department of Emergency Medicine, Indianapolis, Indiana; ¶ University of Michigan Medical School, Department of Emergency Medicine, Ann Arbor, Michigan; || Harvard Medical School, Department of Emergency Medicine, Boston, Massachusetts

Learning is critical to developing and maintaining competence. Learning is slow at the beginning, accelerates rapidly as we gain skills and knowledge, and then slows again as we achieve competence and approach expertise. Rapid periods of expansion of ability and understanding alternate with stages of relative inertia. We may at times focus on routinization, the repetitive effort by which we standardize aspects of our practice, producing a steady practice state that is efficient and systematic. At other times, however, patients and systems demand a more dynamic approach to learning.

This notion of dynamic expertise requires emergency medicine (EM) practitioners to continually adapt; the very nature of EM requires it. If a practitioner does not exert at least some adaptive effort in response to pressures, expertise erodes and, in extreme situations, a competency threshold may be breached.^1^ In practice, maintenance of competency looks similar to evolution – periods of static equilibrium where little adaptive energy is required punctuated by intense periods of exploration or expansion of skills. At no time has this become more evident than during the COVID-19 pandemic.

COVID-19 has disrupted our equilibrium, dictating rapid evolutionary advances in our EM knowledge and skills. The fault lines in our expertise have been laid bare and our individual and organizational adaptability tested to the point of near breaking.^2^–^5^ Systems and individuals alike have had to flex their adaptive expertise in the face of this strain. Emergency physicians rapidly developed new methods of patient assessment, intubation, ventilation, and critical care to name but a few.^6^–^9^ There has been rapid dissemination of innovation, with the worldwide medical community quickly sharing, learning, and adapting to address the crisis on the patient and system levels.^10^ We have developed, out of necessity, a type of expertise in which the EM expert is newly facile with innovation, flexibility, and adaptability.^11^–^13^

Emergency physicians know intuitively that one size does not fit all. Every day brings novelty and complexity. COVID-19 taught us new lessons in adaptive expertise, yet as EM educators we may not think intentionally about training our learners and ourselves in becoming adaptive experts able to maintain competence in the face of disruptive pressures.

To promote the type of adaptive expertise that allows emergency physicians to be innovators and lifelong learners, it is important to teach not just EM facts, skills, and procedures. We also need to provide our EM learners with the mindset and ability to be adaptive.^14^ In other words, our learners should be encouraged to develop “the ability to learn new information, make effective use of resources, and invent new procedures in order to support learning and problem solving in practice.”^15^ The adaptive lens emphasizes learning that occurs with awareness of the complexity of context, and encourages learners to become aware of new features as well as recognizing old features (Table 1).^16^

A number of learning conditions or contexts facilitate a trainee’s preparation for adaptation. Many of these will be familiar to the emergency physician (Table 2). For example, learning from a *wide range of examples* allows for the recognition that although illness scripts may represent the typical case of a given condition, no illness or condition is without variability. “If you’ve seen one case, you’ve seen one case” often rings true. By experiencing not just repetition but *varied* repetition, the adaptive clinician learns how to deal with not just rare cases but also the “not-yet-encountered” variants. The learner understands to trust their instinct, but to be aware of atypical presentations or complexities of illness that require new or adapted approaches to diagnosis or treatment. As faculty we can ask the learners to identify the uniqueness of each patient case and to approach care with flexibility and inquiry.

Further, as we train residents we have a tendency to scaffold their learning, risking keeping them in their comfort zone. To develop adaptive expertise, it can be helpful to pull residents outside their comfort zone, *challenging them to develop new approaches* to situations. Through this process they develop flexibility to match whatever a situation presents.

Another method of optimizing for adaptation is to encourage a *deep mechanistic understanding* of illness to be able to approach new patient presentations. Through this deep understanding the learner may step beyond usual recipes to innovate new approaches. In patients with COVID-19, application of routine ventilation strategies was quickly shown to be inadequate. A mechanistic understanding of pulmonary function allows recognition of potential optimal ventilation strategies and patient positioning when confronted with the striking differences required in COVID-19 management.

Finally, to recognize how to balance adaptation and efficiency, learning must contain *opportunities for application* of each. It does little good to emphasize efficiency only in routine cases or to emphasize innovation only in unusually complex scenarios. As residents focus on patient volume and flow, they may lose their deeper learning of mechanisms, variability, and clinical curiosity. Educators do well to highlight opportunities to innovate even in relatively mundane situations, and to identify opportunities to practice efficiency even in highly complex cases. In this way, EM learners can be positioned in the so-called “optimal adaptability corridor,”^15^ being able to appropriately balance routinization with innovation, a skill unto itself (Figure).

It should come as no surprise that these conditions – a wide range of examples informed by a deep mechanistic understanding and an opportunity to explore both innovation and efficiency – sound familiar to the emergency physician. During COVID-19, EM practice moved from routine to adaptive expertise.

If we can now remain intentional about training for adaptability, it is possible that EM training programs *can be* the shining examples of training for adaptive expertise. As a maturing field, EM has retained its penchant for cutting edge innovation and its deserved reputation for flexibility and adaptability. As we continue to digest the worldwide response to the individual and system stressors brought about during the ongoing pandemic, it is not too soon to begin to celebrate the adaptability that EM has demonstrated. As educators, however, we need to focus on how we will teach for adaptability to ensure our learners are prepared for whatever the next disruptions will be. There must be room in our educational models for both business as usual (and how to do business as usual better), and for exploration beyond the bounds of what is usual. We believe that emergency physicians are well equipped to set the standard for learning the personal and organizational capacity for adaptability.

As we consider the training of future adaptive experts, we must recognize that their expertise will include negotiating the balance between compiled routine expertise (efficiency) and reflective, disruptive and on-demand expertise (innovation), and the ability to identify when to toggle between them (adaptability).^18^ Our training mindset must continue to mirror these processes: nimble, flexible, and responsive to the changing needs of our health systems and our learners. When the system strains under stress, this adaptive expertise becomes not just admirable, but necessary.

## Figures and Tables

**Figure:**
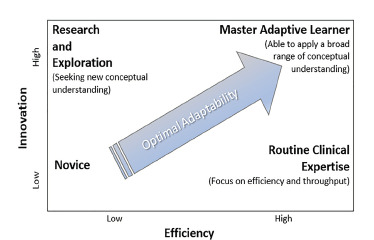
The balance of routine expertise (efficiency) and adaptive expertise (innovation) bound the optimal adaptability corridor (adapted from Bransford et al.^15^).

**Table 1:** A comparison of traditional teaching methodology with teaching for adaptive expertise. Teaching for adaptive expertise may not replace more traditional teaching methods in all cases but ought to be built into emergency medicine training early and often.

Parameter	Traditional method	Teaching for adaptive expertise
Emphasis	Efficient learning of well-known illness scripts and prototypic examples	Developing expertise that can match any variation or situation that is presented
Unit of Adaptation	Environment is adapted to the learner	Learner learns to adapt to the environment
Learning support	Allowing learners to gain full confidence within their comfort zones	Give learners approaches for adapting outside their comfort zones
Progression	Progressive withdrawal of learning supports as learners near competence	Progressive addition of adaptive behaviors
Endpoint	Full withdrawal of learning supports at competence.	No endpoint – coaching long-term for continued improvement, innovation, and adaptation

**Table 2:** Conditions that optimize learning for adaptive expertise.

Conditions for learning adaptive expertise:	Examples:
Learning from a wide range of examples	Exposure to a variety of patient and illness presentations, varying in context and severity, repeated over time
Challenging learners to develop new approaches	Encouraging learners to identify gaps in their understanding and to step beyond their comfort zones, intentionally building, testing, and applying new approaches to even familiar conditions^17^
Encourage deep mechanistic understanding	Returning to first principles when considering how and why a condition may present in varied fashion. Asking “What if...?” and “Why?” when faced with routine problems.
Learning through repeated opportunity for application of both routinization and innovation	Alternately seeking to improve efficiency, apply innovation, and attend to the balance between them. “Is this the wheezing patient that requires a bespoke solution?”
